# Implants Placed with a Ring Technique Using Inlay and Onlay Block Xenografts in the Mandible of Rabbits

**DOI:** 10.3390/ma16237490

**Published:** 2023-12-03

**Authors:** Naoki Kaneko, Samuel Porfirio Xavier, Kenzo Morinaga, Daniele Botticelli, Erick Ricardo Silva, Yasushi Nakajima, Shunsuke Baba

**Affiliations:** 1 Department of Oral Implantology, Osaka Dental University, 8-1 Kuzuhahanazonocho, Hirakata 573-1121, Osaka, Japan; dentalkei@outlook.jp (N.K.); morinaga-k@cc.osaka-dent.ac.jp (K.M.); y.nakajima@me.com (Y.N.); baba-s@cc.osaka-dent.ac.jp (S.B.); 2 Department of Oral and Maxillofacial Surgery and Periodontology, Faculty of Dentistry of Ribeirão Preto, University of São Paulo, Av. do Café-Subsetor Oeste-11 (N-11), Ribeirão Preto 14040-904, SP, Brazil; spx@forp.usp.br (S.P.X.); erickricardo.rp@gmail.com (E.R.S.); 3 ARDEC Academy, 47923 Rimini, Italy

**Keywords:** animal study, bone healing, histology, lateral augmentation, bone transplantation, biomaterial, bone defect, inlay, onlay, xenograft

## Abstract

Background: Xenogenous bone has been proposed as an alternative to overcome the disadvantages of autogenous grafting. The aim of the present study was to study bone dynamics at inlay and onlay xenografts used for bone augmentation applying a ring technique. Methods: The bone at the lateral surface of the mandibular angle of 12 adult male New Zealand White rabbits was exposed bilaterally. The cortical layer received multiple perforations on one side of the mandible, and a xenograft block of collagenated cancellous equine bone, 7 mm in diameter and 3 mm in width, was fixed on the prepared surface using an implant (onlay group). On the opposite side, a defect 7 mm in diameter and 3 mm in depth was prepared, and the xenograft block was adapted to the defect and fixed with an implant (inlay group). Results: After ten weeks of healing, in the onlay grafts, new bone was mainly formed on the trabeculae surface, reaching in some specimens the most coronal regions of the block. In the inlay grafts, new bone was found arranged on the trabecular surfaces but also occupying the spaces among the trabeculae. The entrance of the defect was often found close to the top of the block by newly formed bone. A higher percentage of new bone was found in the inlay (19.0 ± 9.3%) compared to the onlay (10.4 ± 7.4%) groups (*p* = 0.031). The mean gain in osseointegration at the implant in relation to the base of the original 3 mm deep defect was 0.95 ± 1.05% in the onlay group and 0.78 ± 0.71% in the inlay group (*p* = 0.603). Conclusion: The inlay grafts exhibited a higher new bone percentage than the onlay block grafts possibly due to the defect conformation that presented more sources for bone growth. The trabecular conformation and the composition of the grafts made possible the expression of the osteoconductive properties of the material used. This resulted, in several specimens, in the growth of bone on the graft trabeculae toward the most superior regions in both groups and in the closure of the coronal entrance of the defects in the inlay group. The clinical relevance of this experiment is that the ring technique applied as an inlay method could be suitable for bone augmentation.

## 1. Introduction

An atrophied posterior mandible, because of tooth loss, periodontal pathologies, tumor removal, trauma, or congenital diseases, represents a challenge for implant-supported rehabilitation because of the reduction in bone volume between the residual alveolar ridge and the mandibular canal [1,2,3]. Bone augmentation has become an essential component of implant dentistry in this scenario [4,5,6].

The inlay technique has proven to be highly effective for the incorporation of bone grafts, with low resorption over time and high implant survival and success rates [7,8,9]. Although good results have been reported for posterior mandibular bone augmentation using this technique [10,11,12,13], it demands considerable surgical experience, and at least 4 to 6 mm of bone height over the mandibular canal is needed [11,12].

Onlay bone grafts have been successfully used in mandibular bone augmentation. This technique has been associated with high rates of bone resorption before implant placement [2,14]. The soft tissue quality is the primary consideration during the reconstruction of the posterior mandibular region. A lack of keratinized tissue may contribute to wound dehiscence, infection, and subsequent necrosis of the graft [15].

Autogenous bone harvested from either extra- or intraoral donor sites is the gold-standard graft for alveolar ridge augmentation because of its osteoconductive, osteoinductive, and osteogenic properties [16,17,18,19]. Nevertheless, autografts have several disadvantages related to complications at the donor site, limitation of bone surgical morbidity at the donor site, postoperative pain, limited bone disposal, increased surgical time, and financial costs [20,21,22,23,24,25].

To address the drawbacks of autologous grafting, xenogenous bone has been proposed as an alternative [26,27,28]. Using xenogenous bone blocks from both equine and autogenic sources for onlay mandibular augmentation in rabbits, a recent study compared the incorporation and remodeling processes. Although the bone volume and quality of the grafted area were superior for autogenous bone, the amount of new bone formation after 60 days was similar for autologous and xenogeneic bone blocks [29]. More recently, the healing of collagenated equine bone blocks used as either inlay or onlay for mandibular bone augmentation in rabbits has been assessed [30]. The inlay group showed a faster and higher increase in the percentage of newly formed bone.

Installing implants simultaneously with a bone graft can reduce the total treatment time by eliminating a second surgery. Several clinical [31,32,33,34,35,36] and experimental [37,38,39,40,41] studies reported data on implants installed with ring grafts secured by the implant for vertical bone augmentation.

To investigate the efficacy of this approach, a randomized clinical trial evaluated marginal bone loss of autogenic bone blocks collected from the chin used for either inlay or onlay grafting with simultaneous implant placement [42]. Bone gain was significantly higher for the inlay group. However, the comparison between xenogeneic bone blocks used for inlay and onlay grafts with implants has not yet been investigated.

Thus, the aim of the present study was to study bone dynamics at inlay and onlay xenografts used for bone augmentation applying a ring technique.

## 2. Materials and Methods

### 2.1. Ethical Statements

The Committee on Ethics in the Use of Animals of the Faculty of Dentistry of Ribeirão Preto, University of São Paulo, Brazil approved the experiment on 16 October 2019, as reported in protocol 2019.1.694.58.4. The rules reported in the legislation for animal experimentation in Brazil were strictly followed. The ARRIVE Checklist was used in this study.

### 2.2. Study Design

This was a split-mouth, prospective, controlled, randomized study. Xenograft blocks were applied to the lateral wall of the mandible using the onlay or inlay techniques. Both blocks were fixed to the recipient sites using an implant. The healing was evaluated after 10 weeks.

### 2.3. Experimental Animals and Sample Size

Data from a similar study in rabbits were used to determine the sample size [43]. Considering 10% of the difference in the new bone between the two groups as histologically relevant with the maximum standard deviation of 7.4% observed in the study, a sample size of eight animals was obtained to reject the null hypothesis that this response difference is zero by applying α = 0.05 and a power of 0.9 (PS, Dupont and Walton D. Plummer). The sample size was increased to 12 to account for possible complications at various levels during the experiment.

Hence, 12 adult male New Zealand White rabbits weighing 3.5–4.0 kg and aged between 5 and 6 months were included in the study. Upon arrival at the institution, animals were properly identified through tags specifying the cage number, species, weight, and scheduled dates for surgery and euthanasia. Additionally, animals received identification numbers on the inner part of the ear using a permanent pen.

### 2.4. Randomization and Allocation Concealment

An author not involved in animal selection, surgery, and histological evaluation performed the randomization electronically (S.P.X.). The allocation treatment was concealed in opaque sealed envelopes that were opened after recipient site preparation just before the placement of the first xenogeneic block. The histological slides were examined by an assessor (E.F.D.R., see acknowledgements) who was not informed about the allocation treatment. However, the treatment was easily recognized on histological slides.

### 2.5. Biomaterials

**Figure 1:**
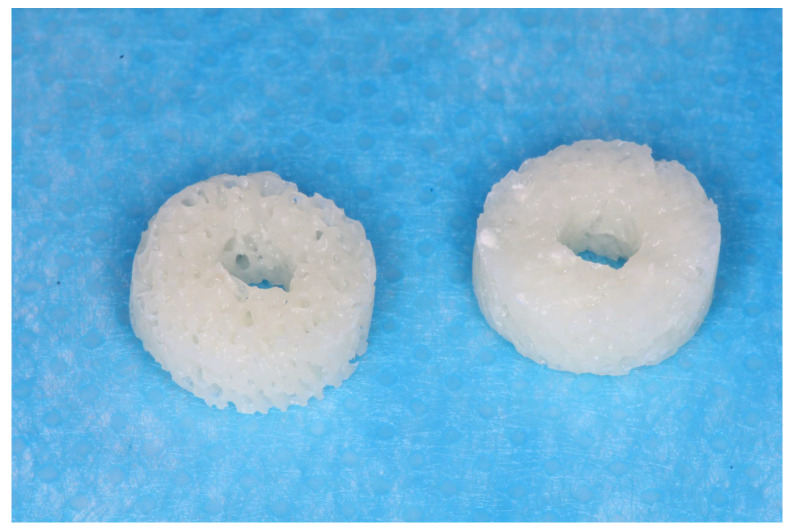
Xenografts with a hole in the center for implant insertion.

SpBlock is a xenograft block of exclusively collagenated cancellous equine bone (Tecnoss, Giaveno, Italy). This process prevents the ceramization of hydroxyapatite crystals, aiming to accelerate resorption. The blocks were cylindrical with diameters of 7 mm and thicknesses of 3 mm. A hole, in the dimensions of the implant, was prepared in the center of the blocks (Figure 1). The central hole in the blocks was created using a sequence of drills at a speed of 800 rpm and 20 N/m under constant irrigation with 0.9% saline solution until a final diameter of 3.3 mm was obtained, corresponding to the diameter of the implant designated for fixing the blocks.

Bio-Gide (Geistlich Biomaterials, Wolhusen, LU, Switzerland) is a porcine-derived resorbable membrane composed of types I and III collagen. The bilayer structure contains an outer smooth layer that prevents the invasion of soft tissues and an inner layer that favors the growth of vessels and cells [44].

### 2.6. Anesthetic Procedures

Anesthetic procedures were carried out using acepromazine (1.0 mg/kg; Acepran, Vetnil, Louveira, São Paulo, Brazil), administered intramuscularly in the hind leg of the animal, and xylazine (3.0 mg/kg; Laboratórios Calier S/A, Barcelona, Spain) combined with ketamine (50.0 mg/kg; União Química Farmacêutica Nacional S/A, Embuguaçú, São Paulo, Brazil), also administered intramuscularly 15 min after the acepromazine administration. Once adequately sedated, the animals underwent prophylactic antibiotic therapy with oxytetracycline (0.2 mL/kg; Biovet, Vargem Grande Paulista, São Paulo, Brazil) administered intramuscularly. Additionally, they received ketoprofen (3.0 mg/kg, 12/12 h, i.m., Ketofen 10%, Merial, Campinas, São Paulo, Brazil) and tramadol hydrochloride 2% (1.0 mg/kg, 12/12 h, subcutaneous, Cronidor, Agener União Saúde Animal, Apucarana, Paraná, Brazil) preoperatively and were maintained postoperatively for the subsequent 3 days.

### 2.7. Surgical Procedure

One qualified expert surgeon performed all surgeries (V.F.B.; see acknowledgments).

**Figure 2:**
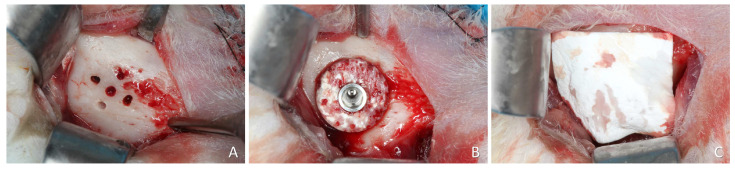
Onlay experimental site. (**A**) Recipient site prepared with perforations. (**B**) Block graft fixed with an implant at the top of the experimental region. (**C**) A collagen membrane placed at the top of the experimental region.

A 2.5–3 mm long incision was made bilaterally on the skin in the lower border of the mandible. The muscles and the periosteum were reflected to expose the lateral surface of the posterior region of the body of the mandible, close to the mandibular angle. At the onlay sites, five calibrated perforations of the cortical layer up to the bone marrow compartment were performed (Figure 2A) using a 1.0 tapered drill (Beavers Jet Burs, Morrisburg, ON, Canada), attached to a straight handpiece, operating at a speed of 20,000 rpm and 20 N/m under constant irrigation with a 0.9% saline solution. This approach was employed to perform five equidistant monocortical perforations, guided by a template crafted from stainless steel. These perforations reached the medullary portion of the recipient site with the purpose of fostering blood and cellular supply to the graft, originating from the endosteal region.

The central perforation was enlarged for implant placement. The xenogeneic bone block was fixed on the prepared region using an implant 8.5 mm long and 3.25 mm in diameter (Leader Medica, Padua, Italy) (Figure 2B). The implant margin was placed at about the level of the graft. A cover screw was positioned on the top of the implant and a collagen membrane was used to cover the experimental region (BioGuide^®^; Geistlich Pharma AG, Wolhusen, Switzerland) (Figure 2C).

**Figure 3:**
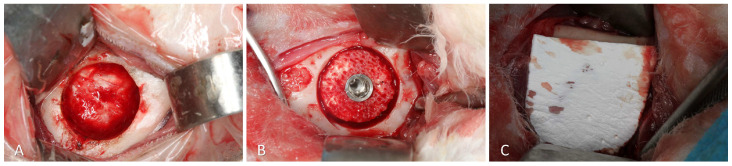
Inlay experimental site. (**A**) Preparation of the 7 mm wide and 3 mm deep defect. (**B**) Placement of the xenograft block inside the defect, secured with an implant. (**C**) Collagen membrane placed on the top of the graft.

Trephines and drills were used on the opposite side to obtain a defect ~7 mm wide and ~3 mm deep. (Figure 3A). The xenogeneic bone block was adapted within the defect at the level of the adjacent bone and fixed with an implant 8.5 mm long and 3.25 mm in diameter (Leader Medica, Padua, Italy) (Figure 3B). After placement of the cover screw, the region was covered with a collagen membrane (BioGuide^®^; Geistlich Pharma AG, Wolhusen, Switzerland) (Figure 3C). The wound closure was performed using Vicryl 4-0 (Ethicon, Johnson & Johnson, Cincinnati, OH, USA) for the muscle layers and Nylon 4-0 (Ethicon, Johnson & Johnson, USA) for the skin, employing simple interrupted sutures.

### 2.8. Animal Maintenance

After surgery and during the following 3 days, the animals received ketoprofen (3.0 mg/kg, 12/12 h, i.m., 10% Ketofen, Merial, Campinas, São Paulo, Brazil) and 2% tramadol hydrochloride (1.0 mg/kg 12/12 h, subcutaneous; Cronidor, Agener União Saúde Animal, Apucarana, Paraná, Brazil).

The rabbits were housed in individual metal cages (1 animal/4500 cm^2^) in an acclimatized room with split air conditioning, an exhaust fan (27 to 34 air changes/h), and automatic lighting control (12-h light-dark cycle) at the Animal Facility of the Faculty of Dentistry of Ribeirão Preto, University of São Paulo. The animals received dedicated food and had ad libitum access to water. Every day, a careful check of the basic biological functions, feeding and excretion, behavioral signs in relation to postoperative pain, and monitoring of post-surgical infections and surgical wounds for suture care, bleeding, and/or signs of infection was performed.

### 2.9. Euthanasia

Animals were euthanized by administering an overdose (2.0 mL) of intravenous thiopental 1.0 g (Thiopentax; Cristália, Itapira, São Paulo, Brazil). Euthanasia was conducted in accordance with the guidelines of the Ethics Committee of the Ribeirão Preto School of Dentistry after a 10-week healing period.

### 2.10. Histological Processing

The experimental regions were dissected, reduced to individual blocks, and fixed in 10% paraformaldehyde for a period of 10 days with regular formaldehyde changes every 2 days. Initially, the specimens were rinsed under running water to ensure complete removal of the fixative agent. Subsequently, they underwent a gradual and ascending dehydration sequence in ethyl alcohol, changed every three days with constant agitation (60%, 80%, 96%, and absolute alcohol twice). Following this, the specimens were immersed in resin (LR WhiteTM HardGrid, London Resin Co., Ltd., Reading, Berkshire, UK) for impregnation, and subsequent polymerization was conducted in an oven at 60 °C.

After polymerization, each block was cut along a transaxial plane at the center of the block, guided by the implant positioned at the center of the graft. Two sections of approximately 100–150 µm were prepared using a precision cutting/grinding instrument (Exakt, Apparatebau, Norderstedt, Germany) and ground until slices with an approximate thickness of 60–80 µm were obtained. The histological sections were stained with Toluidine Blue, Stevenel’s Blue, and Alizarin Red.

### 2.11. Histomorphometric Evaluation

All histomorphometric assessments were performed by an expert assessor (E.F.D.R., see acknowledgements) who did not participate in the other stages of the study, after having compared the results with another expert (D.B.) until inter-rater agreement achieved a Cohen’s coefficient of k > 0.90.

**Figure 4:**
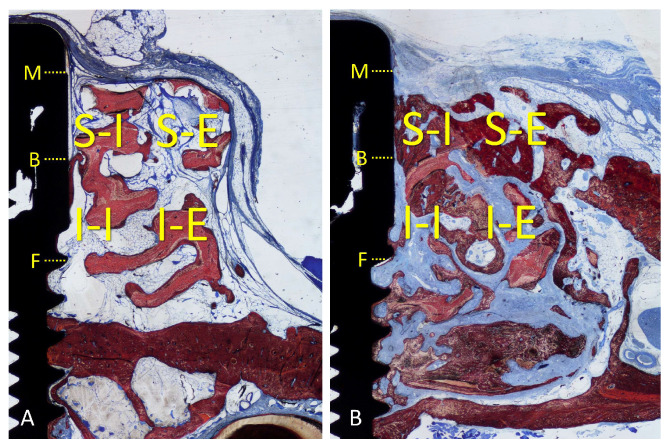
(**A**) Onlay graft; (**B**) inlay graft. Regions evaluated: inferior/internal (I-I), inferior/external (I-E), superior/internal (S-I), and superior/external (S-E). M, implant margin; B, coronal level of osseointegration; F, bottom of the defect.

An Eclipse Ci microscope (Nikon Corporation, Tokyo, Japan) connected to a computer was used for histological assessments by applying a ×10 lens. All histomorphometric assessments were performed with the software NIS Elements D software (v 5.0, Laboratory Imaging, Nikon Corporation, Tokyo, Japan). As a linear evaluation, the distance between the implant margin (M) and the most coronal contact of the bone with the Implant surface (B, coronal level of osseointegration) was measured (Figure 4A,B). The gain of osseointegration was evaluated as the difference between the depth of the original defect (F, 3 mm from M) and the distance M-B. For morphometric measurements, a lattice was superposed on the image by applying point-counting methods. Four regions were evaluated within the grafted region, both sides lateral to the implant: inferior/internal (I-I), inferior/external (I-E), superior/internal (S-I), and superior/external (S-E). The following tissues were assessed: new bone, xenograft, soft tissues (marrow spaces, provisional matrix, dense and loose tissues, and connective tissue), and inflammatory infiltrate (Figure 4A,B). A correlation between the bone percentage in the S-I region and the gain of osseointegration was carried out.

### 2.12. Experimental Outcomes and Statistical Methods

Data are reported as the mean ± standard deviation. The primary variables were the mineralized new bone percentage within the grafts and osseointegration gain. The secondary variables were the other tissues evaluated in the morphometric analysis. Differences between onlay and inlay were evaluated using a paired *t*-test or Wilcoxon matched-pairs signed-rank test. The selection of the test was based on the results of normality assessed by applying the Shapiro–Wilk test. GraphPad Prism (version 10.0.2 for Windows, GraphPad Software, Boston, MA, USA) was used for the statistical analysis. The significance level was 5%.

## 3. Results

### 3.1. Clinical Outcomes

The healing of the animals was uneventful. All histological slides were available for analysis, with n = 12.

### 3.2. Descriptive Histological Evaluation

**Figure 5:**
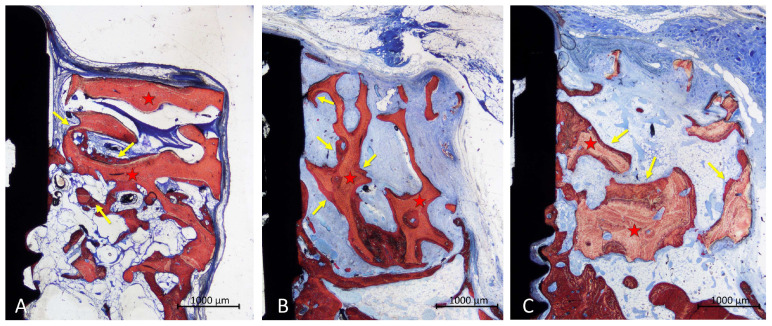
Photomicrographs of ground sections illustrating the healing in the onlay grafts. New bone was mainly found covering the trabeculae (**A**,**B**), in some cases reaching the most coronal regions (**C**). Landmarks indicating examples of tissues: yellow arrows, new bone; red stars, graft. Stevenel’s blue and alizarin red stain.

In the onlay group, new bone was found mainly laying on the trabeculae of the xenograft (Figure 5A,B), in some cases reaching the most superficial region (Figure 5C). The areas within the trabeculae were filled with bone marrow in a few cases (Figure 5A,C) or more often by dense soft tissue (Figure 5B).

The configuration of the trabeculae of the graft seemed to have been maintained in several grafts, whereas in other cases, the trabeculae appeared to have lost the original conformation. An inflammatory infiltrate was observed in only one specimen. Most of the implants showed new bone on the surface of the implant within the defect, in some cases up to a distance of <1 mm from the implant margin (Figure 5C).

**Figure 6:**
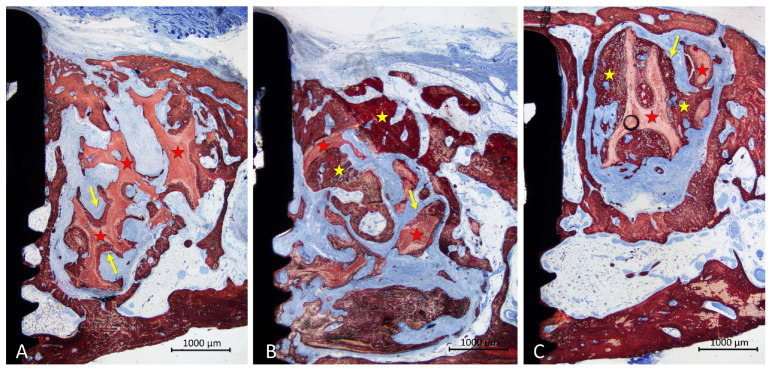
Photomicrographs of ground sections illustrating the healing in the inaly grafts. Bone was found covering the surface of the xenograft but also occupying large spaces within the trabeculae (**A**–**C**). The coronal entrance of the defect was often closed by new bone formed from the margins of the defect and connected by the bone formed within the graft. Landmarks indicating examples of tissues: yellow arrows and stars, new bone; red stars, graft. Stevenel’s blue and alizarin red stain.

In the inlay group, new bone lay on the surface of the trabeculae and occupying the spaces among trabeculae (Figure 6A–C). Dense soft tissue was occupying the remaining spaces among the trabeculae. The coronal entrance of the defect was often found closed by new bone formed from the edges of the defect, interconnected with that formed within the graft (Figure 6A–C). The trabeculae of the graft were still present in the region, however, in different proportions in the various specimens. A small inflammatory infiltrate was observed only in one specimen. Most implants showed new bone formed on the implant surface in the defect region, in some cases up to <1 mm from the implant margin (Figure 6A–C).

### 3.3. Histomorphometric Assessments

**Figure 7:**
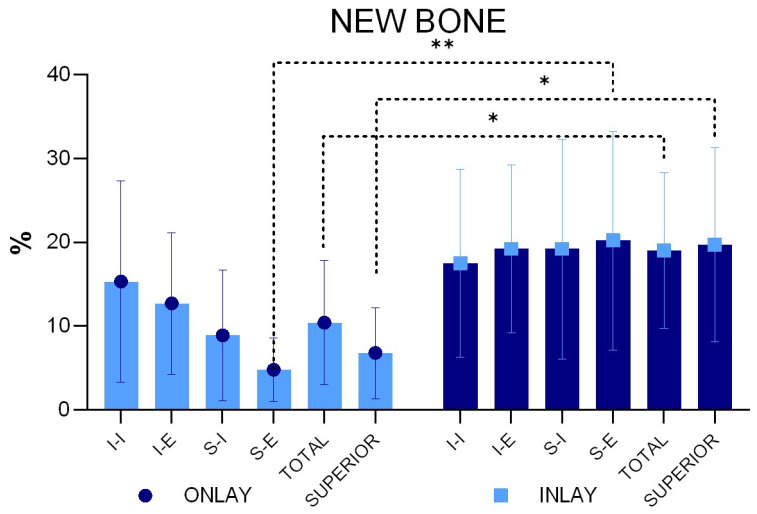
New bone percentages in the various regions examined. * *p* < 0.05; ** *p* < 0.01 (Graphs produced with GraphPad Prism).

After ten weeks of healing, a statistically significantly higher percentage of new bone was found in the inlay compared to the onlay graft, being 19.0 ± 9.3% and 10.4 ± 7.4%, respectively (*p* = 0.031) (Figure 7).

The new bone percentage was higher at the inlay than at the onlay grafts in all four regions examined, with the difference being statistically significant only for the S-E region. When the bone percentages of the two superior regions were merged (mean S-E and S-I), a statistically significant difference was found between the two groups (*p* = 0.010).

**Figure 8:**
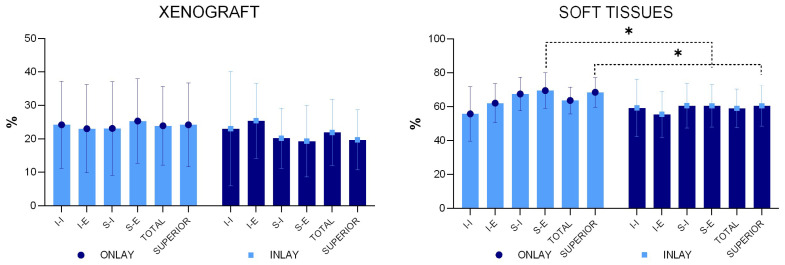
Xenograft and soft tissue percentages in the various regions examined. * *p* < 0.05 (Graphs produced with GraphPad Prism).

The mean percentage of the xenograft did not yield statistically significant differences in any of the regions evaluated. An inflammatory infiltrate was observed only in one specimen of the onlay graft (Figure 8).

Even though, in some cases, the coronal level of osseointegration on the implant was located <1 mm from the implant margin, other sites presented any or very little bone gain on the implant surface above the 3 mm deep original defect. The mean gains were 0.95 ± 1.05% in the onlay group and 0.78 ± 0.71% in the inlay group (*p* = 0.603). The r-value correlation between the new bone percentage in the superior interior region S-I and the level of osseointegration was 0.7 (a strong positive linear relationship).

## 4. Discussion

The current research focused on comparing the bone dynamics of implants installed with inlay or onlay grafts for lateral bone augmentation in the mandible of rabbits. The results demonstrated a higher new bone mean percentage in the inlay graft (19.0 ± 9.3%) compared to the onlay graft (10.4 ± 7.4%; *p* = 0.031). The highest differences in bone formation between the two groups were observed in the region closer to the top of the grafts, i.e., the furthest regions from the bone walls.

The reasons for these differences in the proportion of new bone are to be referred to the different characteristics of the recipient regions. Indeed, inlay grafts were inserted into self-contained defects presenting walls at the base and around the graft. This means that the grafts could rely on bone production from various sources located around them. Instead, the onlay grafts could only rely on bone formed from the cortical layer in contact with the base of the graft. From that recipient site, the new bone had to grow toward the top and the lateral sides of the graft.

In the onlay group, aiming to favor new bone formation, several perforations of the cortical layer were made, reaching the marrow spaces. This procedure has been shown to improve the healing within the graft. In a study on rabbits, the lateral aspect of the mandible angle was prepared with perforations only on one side, while the opposite side was left intact [45]. Block grafts obtained from the iliac crest were secured on the mandibular recipient sites. Revascularization, volume/density maintenance, and the occurrence of bone remodeling proteins were evaluated for different periods of healing. A higher loss of volume was observed in the intact site graft while a higher bone density was measured in the perforated sites. The VEGF labeling became apparent already after 3 days in the perforated group while, in the intact sites, the labeling became evident after 5 days, exhibiting an earlier angiogenesis rate in the former than in the latter groups. The immuno-labeling of osteoblastic lineage showed an accelerated bone remodeling process in the perforated sites. The effect of these perforations was illustrated in detail in another study in which grafts collected from the calvaria were applied on the mandibular angle prepared with perforations [46].

The onlay graft can only count on the osteoconductivity of the biomaterial, which is of foremost importance. Indeed, considering the results from experimental studies, the lack of osteoconductivity of the biomaterial might lead to failures. In a dog study, block grafts composed of deproteinized bovine bone material (DBBM) were placed as onlay on the lateral wall of the alveolar bone in the mandible. A minimal incorporation of the xenograft was observed, limited to the base close to the recipient site [47]. In another experiment in dogs [48], similar DBBM blocks were placed as inlay grafts in defects prepared in the alveolar bone crest in the mandible. On the opposite side of the mandible, an autogenous bone block was used as a graft. While the autogenous bone block presented a perfect incorporation to the recipient site, the xenograft failed to be incorporated, showing, in most cases, a layer of connective tissue interposed between the graft and the recipient sites. Instead, when collagenated blocks were used, new bone was formed within the graft, laying on the trabeculae of the xenograft and reaching the furthest regions from the recipient site [43].

The superior outcome in bone growth of the inlay compared to the onlay block grafts could be related to the positioning of the graft within a self-contained defect that, being delimited by bone walls, offered multiple sources for bone growth. Depending on the dimensions, self-contained defects have the potential to heal spontaneously, similar to the extraction sockets. The spontaneous healing of critical-sized defects was evaluated in rabbits in both the calvaria and mandible [49]. In each animal, through-and-through circumferential defects were prepared, 10 mm in diameter in the calvaria and 11 mm in diameter in the mandible. Autogenous bone or biphasic calcium phosphate granules were placed on the defects of one side of the mandible to fill them out, and the opposite defects were left to heal spontaneously. Both microCT and histological analysis revealed a failure in the closure of the empty defects. The defect applied in the present study presented more favorable conditions compared to the study mentioned above, i.e., smaller dimensions and a box conformation that also allowed bone formation from the base of the defect.

In another experimental study on rabbits [50], 2- to 3-mm-deep box defects of different dimensions (4, 5, 6, 8, and 10 mm) were prepared in the mandible. After 12 weeks of healing, the smallest defects were closed with newly formed bone. However, the 8-mm- and 10-mm-wide defects persisted underfilled. The defects applied in the present study had a box conformation and a diameter of 7 mm. Under such conditions, it is reasonable to assume that a certain degree of spontaneous healing should be expected.

However, an implant was placed in the center of the defect to fix the graft. This implant affected the total volume of the defect and eventually transformed a 7 mm critical-sized defect into a circumferential peri-implant marginal defect with a gap of <2 mm. This allows us to suppose that the placement of an implant might accelerate the healing. In contrast, it has been already demonstrated that the implant delays the healing of marginal defects [51]. In fact, in a marginal defect around an implant, new bone is formed from the lateral walls towards the implant surface and it stops approximately 0.4 mm from the surface, leaving a residual narrow defect around the implant surface, occupied by connective tissues. This defect is closed over time by newly formed bone only if the surface has osteoconductive properties [52]. If the implant surface is not osteoconductive, the residual narrow defect will be not filled by new bone [52,53]. In the present study, an incomplete mean gain of implant osseointegration within the graft region was observed in both the inlay (0.78 mm) and onlay (0.95 mm) groups. In several cases, the osseointegration reached a distance of <1 mm from the implant margin in both groups, showing good osteoconductive properties of the implant surface. The low mean gain of osseointegration is due to a large variability of the results, the reasons for which could be attributed to an insufficient new bone content in the internal regions of some grafts, especially in the superior-internal region. In the absence of bone close to the implant surface, the gain in osseointegration is hampered. This assumption is substantiated by the strong positive linear correlation between osseointegration gain and the percentage of new bone in the superior-internal region.

In another comparable experiment on rabbits, a similar pattern of bone formation was observed as in the present study [30]. In that study, the onlay and inlay xenografts were of the same nature as those used in the present study. However, the grafts were secured on the recipient sites with a fixation screw. Two periods of healing were analyzed, i.e., 2 and 10 weeks. The results showed a higher percentage of new bone at the inlay compared to the onlay grafts. The percentage of the xenograft decreased by approximately one-third between the two periods of healing. In the present study, the evaluation was performed after only 10 weeks of healing. However, with the percentage of the residual grafts after 10 weeks being similar between the two studies, it might be supposed that a similar percentage of graft resorption occurred in both studies.

In the present study, complete closure of the coronal entrance of the defects was observed in several specimens of the inlay blocks. This agrees with similar observations reported in the study mentioned above [30].

The present study adopted a “ring” technique with immediate implant installation. This technique was used for vertical augmentation, the results of which have been reported in systematic reviews of both clinical [54] and animal [55] studies.

The animal model used is a limitation of the present study so the data should be interpreted with caution. Several limitations of the study should be considered, such as the faster rate of healing in rabbits compared to humans, as shown for implant osseointegration [56]. Moreover, the healing of critical defects in the body and the angle of the mandible is hampered by the growth of soft tissue in the region [57] and limited blood supply [58]. These conditions might have compromised healing.

Nevertheless, the experimental studies only reveal possible outcomes that should be considered when similar procedures are applied in humans, and eventually confirmed or refuted. Longer periods of healing should be analyzed.

## 5. Conclusions

The inlay grafts exhibited a higher new bone percentage than the onlay block grafts possibly due to the defect conformation that presented more sources for bone formation. The trabecular conformation and the composition of the grafts made possible the expression of the osteoconductive properties of the material used. This resulted, in several specimens, in the growth of bone toward the most superior regions in both grafts and in the closure of the coronal entrance of the defects in the inlay group. The clinical relevance of this experiment is that the ring technique applied as an inlay method could be suitable for bone augmentation. However, in clinical practice, a vertical defect rarely presents conditions that allow for performing a purely inlay technique. In the best conditions, an intermediate situation between inlay and onlay can be encountered. It is therefore important to model the recipient region in a way that brings this region as close as possible to a self-contained defect. Some xenograft blocks have been shown to have poor osteoconductive properties, making graft selection of critical importance. Furthermore, even if the xenografts have the same conformation as spongy bone, it is necessary for these pseudo-bony trabeculae to be reabsorbed to make room for newly formed bone trabeculae. Simply forming bone on the surface of the xeno-trabeculae cannot be considered optimal healing. This, in turn, means that further studies applying grafts of different materials and conformations are needed to improve outcomes, especially when the grafts are used as onlay.

## Data Availability

The data are available following a reasonable request.
